# Corneal Fibroblasts as Sentinel Cells and Local Immune Modulators in Infectious Keratitis

**DOI:** 10.3390/ijms18091831

**Published:** 2017-08-23

**Authors:** Ken Fukuda, Waka Ishida, Atsuki Fukushima, Teruo Nishida

**Affiliations:** 1 Department of Ophthalmology, Kochi Medical School, Nankoku City 783-8505, Japan; wakai@kochi-u.ac.jp (W.I.); fukusima@kochi-u.ac.jp (A.F.); 2 Department of Ophthalmology, Yamaguchi University Graduate School of Medicine, Ube City, Yamaguchi 755-8505, Japan; tnishida@yamaguchi-u.ac.jp; 3 Ohshima Eye Hospital, Fukuoka City 812-0036, Japan

**Keywords:** fibroblast, keratocyte, cornea, lipopolysaccharide, bacteria, chemokine, adhesion molecule, collagen, tear fluid

## Abstract

The cornea serves as a barrier to protect the eye against external insults including microbial pathogens and antigens. Bacterial infection of the cornea often results in corneal melting and scarring that can lead to severe visual impairment. Not only live bacteria but also their components such as lipopolysaccharide (LPS) of Gram-negative bacteria contribute to the development of inflammation and subsequent corneal damage in infectious keratitis. We describe the important role played by corneal stromal fibroblasts (activated keratocytes) as sentinel cells, immune modulators, and effector cells in infectious keratitis. Corneal fibroblasts sense bacterial infection through Toll-like receptor (TLR)–mediated detection of a complex of LPS with soluble cluster of differentiation 14 (CD14) and LPS binding protein present in tear fluid. The cells then initiate innate immune responses including the expression of chemokines and adhesion molecules that promote the recruitment of inflammatory cells necessary for elimination of the infecting bacteria. Infiltrated neutrophils are activated by corneal stromal collagen and release mediators that stimulate the production of pro–matrix metalloproteinases by corneal fibroblasts. Elastase produced by *Pseudomonas aeruginosa* (*P. aeruginosa*) activates these released metalloproteinases, resulting in the degradation of stromal collagen. The modulation of corneal fibroblast activation and of the interaction of these cells with inflammatory cells and bacteria is thus important to minimize corneal scarring during treatment of infectious keratitis. Pharmacological agents that are able to restrain such activities of corneal fibroblasts without allowing bacterial growth represent a potential novel treatment option for prevention of excessive scarring and tissue destruction in the cornea.

## 1. Introduction

The cornea is located on the external surface at the front of the eyeball and differs from most other tissues in that it is transparent and avascular, properties that allow it to contribute to ocular refraction. The cornea has a relatively simple structure consisting of three layers: the epithelium, stroma, and endothelium with each layer consisting of a different type of structural cell—epithelial cells, keratocytes, and endothelial cells, respectively. Given its location, the cornea is frequently exposed to external insults including microbes, antigens, and inflammatory mediators.

Worldwide, bacterial keratitis is a major cause of visual disturbance and blindness as a result of the corneal melting and scarring that occur if the infection is not treated promptly and appropriately. The Gram-negative bacterium, *Pseudomonas aeruginosa* is one of the most common isolates from individuals with microbial keratitis, especially those who use extended-wear contact lenses [1,2,3]. Not only live bacteria but also their components or products, including the lipopolysaccharide (LPS, also known as lipoglycan or endotoxin) of Gram-negative bacteria, are able to initiate keratitis and subsequent corneal damage [4]. Given that the cornea is an avascular tissue and contains few immune cells, corneal resident cells function as sentinel cells as well as immune modulators during corneal inflammation [5,6,7]. Whereas the corneal epithelium serves as an effective barrier to protect the eye from external agents, corneal stromal fibroblasts (activated keratocytes) play a key role in the recruitment of inflammatory cells into the cornea during acquired or innate immune responses. We previously showed that corneal fibroblasts, but not corneal epithelial cells, recognize the T helper 2 (Th2) cytokines interleukin (IL)-4 and IL-13 in tear fluid [8] and express the chemokines eotaxin (CCL11) [9,10] and thymus- and activation-regulated chemokine (CCL17) [11] as well as vascular cell adhesion molecule-1 in response to such recognition [9,12]. Corneal fibroblasts may thus play an important role in the corneal inflammation associated with severe ocular allergic diseases by promoting the recruitment of eosinophils (via CCL11) and Th2 cells (via CCL17) to the cornea [5,6].

Corneal fibroblasts also recognize bacterial components such as LPS through Toll-like receptors (TLRs) expressed on their surface and thereby activate appropriate innate immune responses in bacterial keratitis. In this review, we will address the role of corneal fibroblasts in LPS-related ocular surface inflammation.

## 2. Innate Immune Responses to LPS in Corneal Cells

LPS is a component of the cell membrane in Gram-negative bacteria and is a potent secretagogue for various cytokines produced by inflammatory cells. Among various TLRs, TLR4 recognizes LPS of Gram-negative bacteria [13]. Although human corneal epithelial cells have been shown to express TLR4, its role in such cells has been unclear. Whereas some studies found this receptor to be functional in corneal epithelial cells [14], others reported that it is expressed intracellularly and therefore does not confer sensitivity to LPS, with the consequent “immunosilent environment” preventing unnecessary responses to commensal bacteria [15]. Although LPS is anchored in the outer membrane of bacteria, it is released spontaneously during bacterial growth. LPS released into tear fluid is not able to stimulate stromal fibroblasts unless the barrier function of the corneal epithelium is compromised. Thus, it is thought to enter the corneal stroma by diffusion only at sites of epithelial abrasion [16].

**Figure 1:**
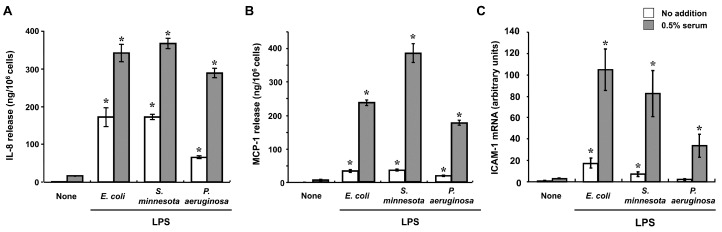
Effects of lipopolysaccharide (LPS) on chemokine release and the abundance of ICAM-1 mRNA in human corneal fibroblasts. Cells were incubated for 24 h (**A**,**B**) or 6 h (**C**) with or without LPS (10 ng/mL) from *Escherichia coli* (*E. coli*), *Salmonella minnesota* (*S. minnesota*), or *Pseudomonas aeruginosa* (*P. aeruginosa*) as well as in the absence (open bars) or presence (closed bars) of 0.5% human serum. The amounts of IL-8 (**A**) and MCP-1 (**B**) released into the culture medium were then determined by enzyme-linked immunosorbent assays, and the amount of ICAM-1 mRNA in the cells (**C**) was determined by reverse transcription and real-time polymerase chain reaction analysis. Data are means ± standard error of the mean (SEM) of quadruplicates from representative experiments. * *p* < 0.05 versus the corresponding value for cells incubated in the absence of LPS. Reprinted with permission from [19].

Injection of LPS into the corneal stroma induced rapid infiltration of inflammatory cells including neutrophils and monocytic cells into the stroma and led to the development of corneal ulceration in rabbits [17,18]. On the basis of these observations, we hypothesized that stromal resident fibroblasts recognize the presence of LPS and trigger inflammatory cell infiltration through expression of chemokines and adhesion molecules. We found that human corneal fibroblasts express TLR4, cluster of differentiation (CD)14, and MD-2, that these proteins form functional LPS receptors [19], and that the cells initiate innate immune responses to LPS through activation of this receptor complex. Corneal fibroblasts produce cytokines and chemokines including IL-6, monocyte chemotactic protein (MCP) 1 (CCL2), and IL-8 (CXCL8), but not IL-1β or tumor necrosis factor-α (TNF-α), in response to LPS (Figure 1). LPS-stimulated corneal fibroblasts also express intercellular adhesion molecule (ICAM) 1 (also known as CD54) [19,20]. Given that the chemotactic activities of MCP-1 and IL-8 as well as adhesion to ICAM-1 expressed on the surface of structural cells mediate the local infiltration and activation of monocytes and neutrophils, the activation of corneal fibroblasts by direct stimulation with LPS may be an important step in the pathogenesis of bacterial infection in the cornea. The cytokine IL-1β plays an essential role in bacterial clearance as well as in the recruitment of neutrophils into the cornea during infectious keratitis [21,22]. However, IL-1β is secreted predominantly by infiltrated inflammatory cells—in particular, by neutrophils themselves—rather than by resident cells. The mechanisms of cleavage and activation of pro–IL-1β are thought to differ between murine models of *P. aeruginosa* keratitis and *Streptococcus pneumoniae* keratitis [21,22]. Infection with *P. aeruginosa* stimulates the production of IL-1β by cultured corneal fibroblasts in a manner that is largely dependent on the sensing of extracellular flagellin of the bacteria by TLR5 [23].

**Figure 2:**
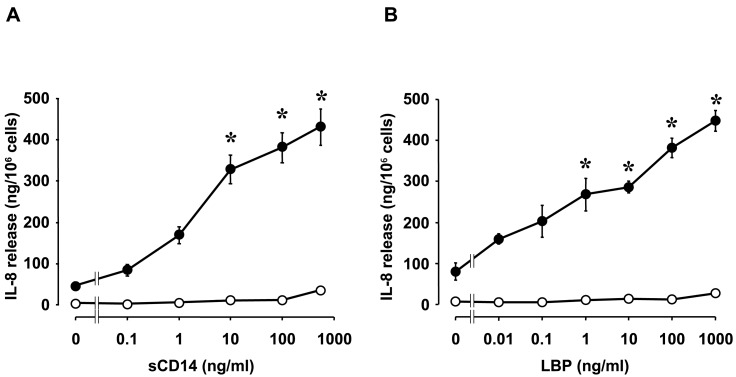
Concentration-dependent effects of soluble cluster of differentiation 14 (sCD14) and LPS binding protein (LBP) on IL-8 release by human corneal fibroblasts. Cells deprived of serum for 24 h were incubated for 24 h in the absence (open circles) or presence (closed circles) of LPS (10 ng/mL) and with the indicated concentrations of sCD14 (**A**) or LBP (**B**), after which the amount of IL-8 released into the culture medium was determined. Data are means ± SEM of triplicates from representative experiments. * *p* < 0.01 versus the corresponding value for cells incubated with LPS in the absence of sCD14 or LBP. Reprinted with permission from [24].

**Figure 3:**
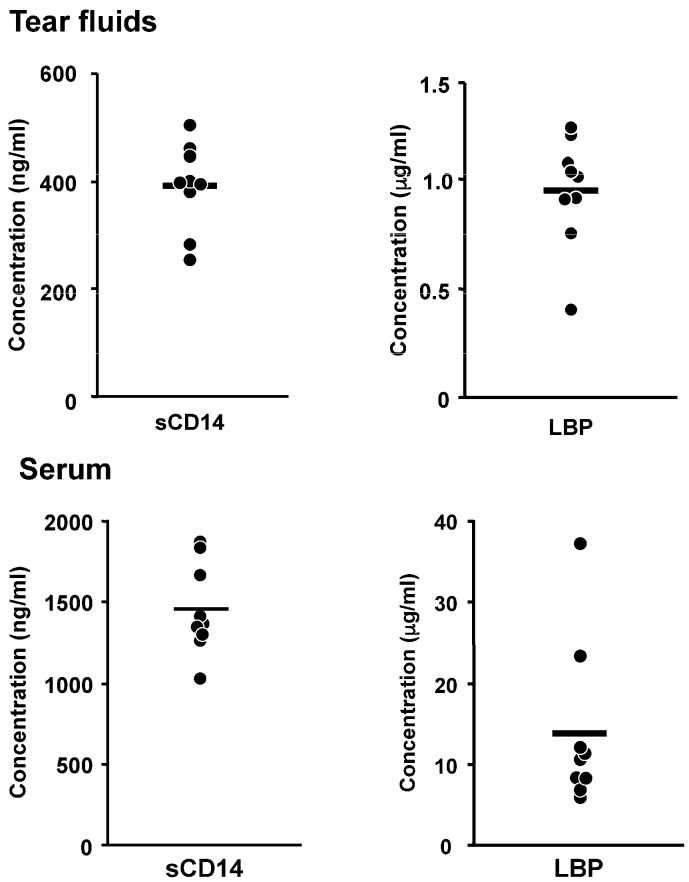
Concentrations of sCD14 and LBP in human tear fluid and serum from the same individuals. Circles, individual values; bars, mean values. Reprinted with permission from [31].

The various effects of LPS on corneal fibroblasts were found to be potentiated by the presence of a low concentration of human serum (Figure 1) [19], suggesting that factors in serum may contribute to the activation of these cells by LPS. Several serum proteins and lipids bind to LPS, and we found that two serum-derived soluble factors—LPS binding protein (LBP) and soluble CD14 (sCD14)—potentiate LPS-induced innate immune responses in corneal fibroblasts [24]. LPS is an amphipathic molecule with a small hydrophilic domain and a large hydrophobic component [25]. Lipid A, the lipophilic portion of LPS, is necessary for endotoxic activity and is highly conserved structurally [26]. In an aqueous environment such as tear fluid, LPS forms polymeric aggregates with the lipid A region facing inward and the hydrophilic polysaccharide component facing outward [25]. Polymeric forms of LPS bind poorly to cells and fail to provoke responses at low concentrations [27]. LBP is produced mostly by the liver and was initially identified as an acute-phase reactant in serum. LBP binds LPS and renders it monomeric, thereby exposing the active lipid A moiety. LBP thus facilitates detection of LPS by its receptors expressed on corneal fibroblasts [18,28,29]. CD14 exists in two forms: a glycophosphatidylinositol-anchored membrane-bound form (mCD14), and a soluble form. Various cell types including inflammatory cells as well as human corneal fibroblasts constitutively express mCD14 at the cell surface, whereas sCD14 forms a complex with LPS that is thought to bind to mCD14-negative cells and thereby to confer sensitivity to LPS. LPS-induced innate immune responses including the expression of chemokines and adhesion molecules in corneal fibroblasts are enhanced by the addition of either LBP or sCD14 (Figure 2) [24]. Although the cornea lacks blood vessels, soluble serum factors are present in the tear fluid that covers the ocular surface. We and others have shown that the tear fluid of healthy adults contains both sCD14 and LBP (Figure 3) [30,31] and our in vitro experiments suggest that they are present at concentrations sufficient to support maximal effects of LPS on innate immune responses in corneal fibroblasts.

Taken together, these various observations suggest that sCD14 and LBP in tear fluid bind LPS and enhance the perception of LPS by corneal fibroblasts, thereby contributing to the first line of immune defense of the cornea against microorganisms.

The innate immune recognition of intracellular LPS by a TLR4-independent mechanism was recently uncovered [32,33]. The lipid A moiety of LPS, when present in the cytoplasm, was thus found to trigger noncanonical inflammasome activation that results in the activation of caspase-11, pyroptosis, and the proteolytic processing of pro-IL-1β and pro-IL-18. However, the presence of this intracellular LPS-sensing pathway and its potential role in corneal fibroblasts remains to be elucidated.

## 3. Therapeutic Intervention Targeting the Role of Corneal Fibroblasts in Infectious Keratitis

The treatment for individuals with bacterial keratitis is administration of appropriate antibiotics. However, LPS is rapidly released from bacteria as a consequence of antibiotic therapy [34], and once corneal fibroblasts have been activated by such released LPS, antibiotics are not able to influence the inflammatory responses of these cells. Although such responses by corneal fibroblasts are the first line of immune defense in the avascular cornea and are important for protection of the host from pathogens at the early stage of infection, persistent and excessive inflammatory responses result in the destruction of corneal tissue.

Keratocytes contribute to maintenance of corneal stromal structure by synthesizing and degrading stromal extracellular matrix proteins including collagen under physiological conditions. Such degradation of the stromal matrix is mediated by matrix metalloproteinases (MMPs) derived from the cells [35,36]. Under pathological conditions such as bacterial infection, the interaction of corneal fibroblasts with invading bacteria and infiltrated neutrophils leads to excessive degradation of stromal collagen. The results of in vitro experiments in which corneal fibroblasts are cultured in a three-dimensional collagen gel suggest that factors including IL-1 secreted by collagen-stimulated neutrophils augment collagen degradation by corneal fibroblasts through a stimulatory effect on pro-MMP synthesis [37]. During infection with *P. aeruginosa*, pseudomonal elastase both degrades type I collagen directly and promotes collagen degradation by corneal fibroblasts through the activation of pro-MMPs released from the fibroblasts [38]. The uncontrolled and prolonged activation of corneal fibroblasts by inflammatory cells or infectious pathogens may therefore lead to destruction of the corneal stroma and corneal scarring. Given the important role of the cornea in ocular refraction, such scarring of the cornea directly results in a loss of vision. It is therefore important that, regardless of the causative factors, corneal inflammation be treated in such a manner as to ensure minimal scarring. Given that infiltration of inflammatory cells into the avascular cornea is regulated by chemokines released by corneal fibroblasts and that stromal collagen degradation is mediated by MMPs produced by these cells, the targeting of stromal fibroblast function is a potential approach to the treatment of corneal inflammation [7]. Although corticosteroids are potent immunosuppressants and attenuate both collagen degradation by corneal fibroblasts as well as the infiltration of mononuclear cells into the cornea in a rabbit model of LPS-induced keratitis [17,39], steroids also promote the proliferation of infecting pathogens. There are currently no eyedrops clinically available for the treatment of infectious keratitis that are able to suppress an excessive inflammatory response without adverse effects. 

Given that activation of nuclear factor–κB (NF-κB) is a key step in the LPS-induced expression of chemokines and adhesion molecules in corneal fibroblasts [19], drugs that are able to inhibit the NF-κB signaling pathway in these cells might be expected to limit the infiltration of immune cells into the cornea. Triptolide, which is present in extracts of the Chinese herb *Tripterygium wilfordii* hook f, possesses anti-inflammatory activity for various cell types including immune cells and tissue resident cells. Triptolide inhibits the activation of NF-κB and thereby attenuates both LPS-induced chemokine and adhesion molecule expression in as well as collagen degradation by human corneal fibroblasts [20,40]. 

**Figure 4:**
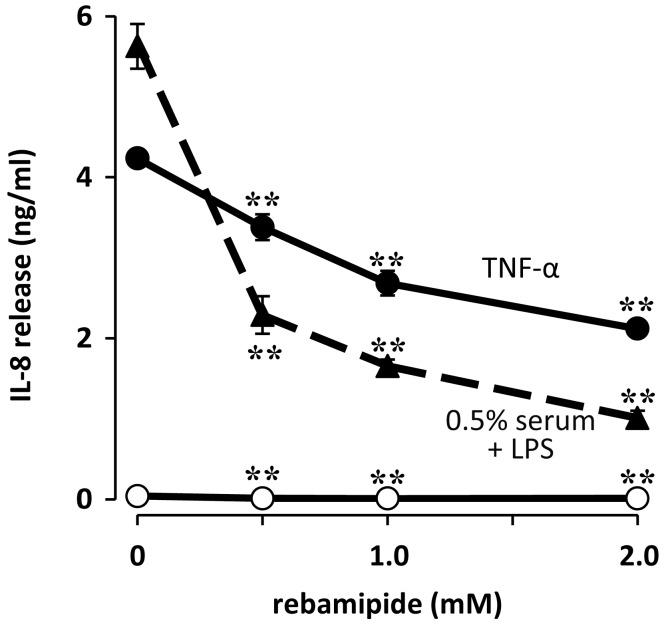
Effect of rebamipide on IL-8 release induced by LPS in human corneal fibroblasts. Cells were incubated first for 1 h with the indicated concentrations of rebamipide and then for 48 h in the additional absence (open circles) or presence either of LPS (100 ng/mL, triangles) plus 0.5% human serum or of tumor necrosis factor-α (TNF-α) (10 ng/mL, closed circles), after which the concentration of IL-8 in culture supernatants was determined. Data are means ± SEM of quadruplicates from a representative experiment. ** *p* < 0.01 versus the corresponding value for cells incubated without rebamipide. Reprinted with permission from [42].

Rebamipide eyedrops were recently introduced to the Japanese market for the treatment of dry eye on the basis of the mucin secretagogue activity of this drug. Rebamipide also manifests various anti-inflammatory effects on corneal epithelial cells and gastric epithelial cells. We recently showed that rebamipide increases the barrier function of human corneal epithelial cells, attenuates the loss of such barrier function induced by the proinflammatory cytokine TNF-α, and inhibits the TNF-α–induced expression of IL-6 and IL-8 in these cells [41]. Rebamipide also suppresses the LPS-induced synthesis of IL-8 through inhibition of NF-κB signaling in human corneal fibroblasts (Figure 4) [42]. Rebamipide may therefore prove effective for the treatment of not only dry eye–related epitheliopathy, but also corneal stromal inflammation associated with bacterial infection or allergy.

A small peptide derived from human pancreatitis-associated protein [43] and an 11-amino acid peptide (RNPRGEEGGPW) derived from hepatocyte growth factor [44] were both found to inhibit the LPS-induced expression of chemokines such as IL-8 and MCP-1 as well as the adhesion molecule ICAM-1 in corneal fibroblasts by preventing NF-κB activation. These peptides also attenuated the corneal inflammation associated with LPS-induced keratitis in mice. In addition, an inhibitor of hyaluronic acid synthesis, 4-methylumbelliferone, as well as hyaluronic acid of high molecular weight were each shown to attenuate the LPS-induced up-regulation of the expression of inflammatory cytokines including IL-6 and IL-8 in rabbit corneal fibroblasts [45]. Furthermore, leukocyte infiltration into the cornea associated with LPS-induced keratitis was found to be restrained in mice deficient in urokinase-type plasminogen activator (uPA) compared with wild-type mice, and the LPS-induced production of both chemokines and MMP-9 was attenuated in corneal fibroblasts from the u-PA-deficient mice compared with those from wild-type mice. These results suggest that targeting of uPA in corneal fibroblasts may inhibit LPS-induced corneal inflammation through down-regulation of chemokine production [46].

Endogenous antimicrobial peptides play a key role in defense against infection at the ocular surface. These molecules are essentially small cationic peptides with broad-spectrum antimicrobial activity against bacteria, fungi, and viruses. Defensins and cathelicidins are two major categories of mammalian antimicrobial peptides and are present at the ocular surface [47,48]. At the ocular surface, α-defensins including human neutrophil peptides 1 to 3 are derived mostly from infiltrating neutrophils, whereas β-defensins such as human β-defensins 1 to 3 are synthesized and secreted by corneal or conjunctival epithelial cells. LL37 is the only member of the cathelicidin family in humans and is secreted by corneal epithelial cells and fibroblasts [49]. The expression of antimicrobial peptides in epithelial cells at the ocular surface is up-regulated in response to bacterial infection through TLRs. TLR2 activation by peptidoglycan or lipopeptide from *Staphylococcus aureus* enhanced the production of LL37 and human β-defensin-2 in corneal epithelial cells [50,51]. LPS from *P. aeruginosa* was also found to stimulate the expression of human β-defensin–2 in corneal and conjunctival epithelial cells via activation of TLR4 [52]. In addition, *P. aeruginosa* flagellin induced the production of human β-defensin–2 and LL37 by corneal epithelial cells through the activation of TLR5 [53]. Several peptides with antimicrobial properties have been tested in vitro as well as in in vivo models for their potential either alone or in combination with antibiotics to treat infectious keratitis [54].

**Figure 5:**
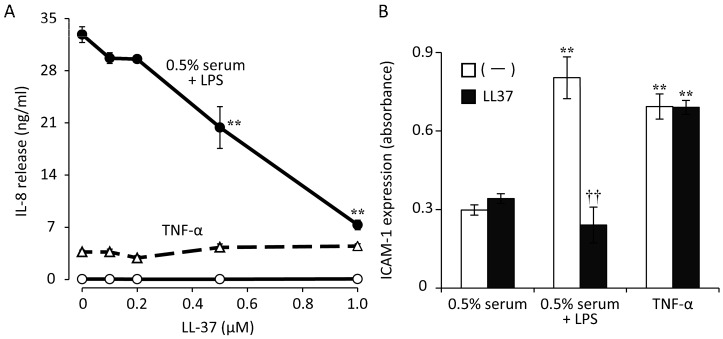
Effects of LL37 on LPS- or TNF-α–induced IL-8 and ICAM-1 expression in human corneal fibroblasts. Cells were incubated first for 2 h with the indicated concentrations of (**A**) or 1 µM (**B**) LL37 and then for 48 h (**A**) or 24 h (**B**) in the additional presence of LPS (100 ng/mL) plus 0.5% human serum (closed circles), of 0.5% human serum alone (open circles), or of TNF-α (10 ng/mL, triangles). The concentration of IL-8 in culture supernatants (**A**) and the cell surface expression of ICAM-1 (**B**) were then determined. ** *p* < 0.01 versus the corresponding value for cells incubated without LL37 (**A**). ** *p* < 0.01 versus the corresponding value for cells incubated with 0.5% serum alone; †† *p* < 0.01 versus the corresponding value for cells incubated without LL37 (**B**). Reprinted with permission from [56].

**Figure 6:**
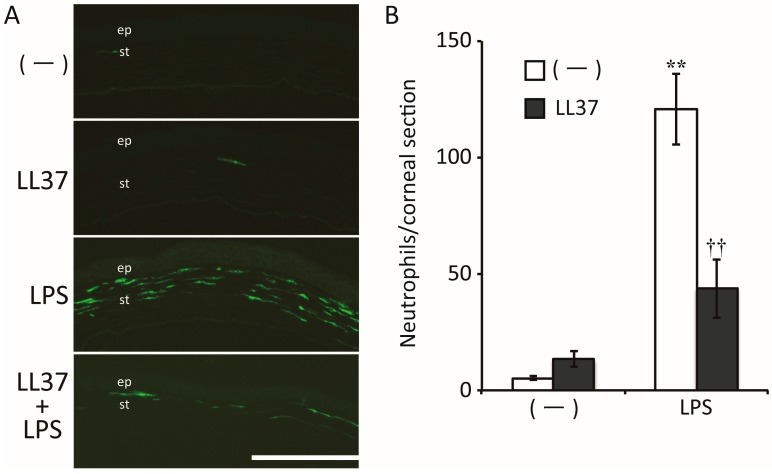
Effects of LL37 in a mouse model of LPS-induced keratitis. (**A**) The cornea was scratched and either LL37, LPS, both LPS and LL37, or phosphate-buffered saline (PBS) vehicle was applied. After 24 h, the eye was enucleated for immunohistofluorescence staining of neutrophils in the cornea. The corneal epithelium (ep) and stroma (st) are indicated. Scale bar, 200 µm; (**B**) the number of infiltrating neutrophils in the corneal stroma in images similar to those in (**A**) was counted. Data are means ± SEM for one section examined for each of four eyes. ** *p* < 0.01 versus the value for PBS alone; †† *p* < 0.01 versus the value for LPS alone. Reprinted with permission from [56].

Drugs that are able to suppress immune responses as well as kill bacteria are also good candidates for the treatment of infectious keratitis. In addition to their direct antimicrobial action, recent studies have revealed that some antimicrobial peptides have pleiotropic effects on various cell types [55]. Among such antimicrobial peptides present at the ocular surface in humans, LL37 and angiogenin have been found to have anti-inflammatory effects on corneal fibroblasts. LL37 reportedly stimulates corneal epithelial wound healing as well as cytokine synthesis by corneal epithelial cells [55]. We have shown that LL37 directly suppresses LPS-induced innate immune responses in corneal fibroblasts (Figure 5), in contrast to its action in corneal epithelial cells. LL37 thus inhibited the expression of IL-6, IL-8, and ICAM-1, as well as the activation of NF-κB, induced by LPS in corneal fibroblasts, whereas it did not attenuate such effects elicited by TNF-α. These inhibitory effects of LL37 on cytokine and adhesion molecule expression thus appeared not to be attributable to nonspecific suppression of NF-κB signaling but rather to be mediated by the binding of LL37 to LPS or to CD14 at the cell surface [56]. We have also shown that exogenous LL37 did not induce corneal inflammation but instead significantly suppressed LPS-induced keratitis in mice (Figure 6). Taken together, these observations suggest that administration of LL37 may be beneficial for the treatment of infectious keratitis on the basis of both its inhibitory effects on corneal fibroblasts and its antimicrobial and pro-wound healing activities. 

Angiogenin is a pro-angiogenic molecule but also acts as an antimicrobial peptide in tear fluid [57,58]. In addition, angiogenin was shown to inhibit the LPS-induced production of IL-6, IL-8, MCP-1, MCP-2, and TNF-α by corneal fibroblasts [59]. Angiogenin may therefore also have a beneficial action in infectious keratitis as a result of its antimicrobial activity and its attenuation of innate immune responses of corneal fibroblasts.

## 4. Conclusions

**Figure 7:**
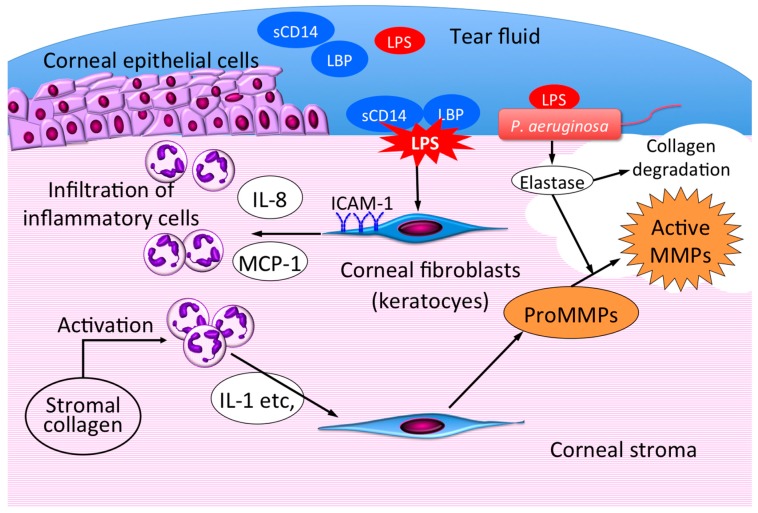
Role of corneal fibroblasts in bacterial keratitis. LPS released from bacteria binds to LBP and sCD14 in tear fluid, and the LPS-LBP-sCD14 complex then activates corneal fibroblasts. The activated fibroblasts promote inflammatory cell recruitment through expression of chemokines and adhesion molecules. Infiltrated neutrophils are activated by corneal stromal collagen and secrete inflammatory mediators including IL-1 that stimulate pro-MMP secretion by fibroblasts. The released pro-MMPs are activated by bacterial proteases such as *Pseudomonas aeruginosa* elastase, and the active MMPs then degrade stromal collagen, leading to corneal melting.

The studies described in this review highlight the central role of corneal fibroblasts in the development of corneal inflammation during infectious keratitis (Figure 7). Corneal fibroblasts sense bacterial infection through the detection of LPS by TLR with the assistance of sCD14 and LBP in tear fluid. Such detection of LPS by corneal fibroblasts triggers innate immune responses including the expression of chemokines and adhesion molecules that promote the recruitment of inflammatory cells, mostly neutrophils, and the consequent elimination of infecting bacteria. Appropriate resolution of infectious inflammation may be prevented, however, by the prolonged overproduction of such inflammatory mediators by corneal fibroblasts, leading to tissue remodeling or destruction and, eventually, to corneal stromal scarring. Infiltrated neutrophils are activated by corneal stromal collagen and release factors including IL-1 that stimulate the production of pro-MMPs by corneal fibroblasts. Certain proteases such as elastase produced by *P. aeruginosa* activate these released pro-MMPs and thereby promote stromal collagen degradation. Corneal fibroblasts thus act as sentinel cells, immune modulators, and effector cells in infectious keratitis. Modulation of corneal fibroblast activation and of the interaction of these cells with inflammatory cells or infecting bacteria is therefore critical to minimize corneal scarring during treatment of infectious keratitis. Drugs that are able to restrain these activities of corneal fibroblasts are needed to expand and improve the treatment options available for infectious keratitis. Pharmacological agents described in this review may provide such novel treatment options to prevent excessive scarring and tissue destruction in the cornea by regulating corneal fibroblast function.

## Abbreviations

LPS	Lipopolysaccharide
Th2	T helper 2
IL	Interleukin
TLR	Toll-like receptor
CD	Cluster of differentiation
MCP	Monocyte chemotactic protein
TNF-α	Tumor necrosis factor-α
ICAM-1	Intercellular adhesion molecule-1
LBP	Lipopolysaccharide binding protein
sCD14	Soluble CD14
mCD14	Membrane-bound CD14
MMP	Matrix metalloproteinase
NF-κB	Nuclear factor-κB
uPA	Urokinase-type plasminogen activator
